# Efficacy and Tolerability of Different Interventions in Children and Adolescents with Attention Deficit Hyperactivity Disorder

**DOI:** 10.3389/fpsyt.2017.00229

**Published:** 2017-11-13

**Authors:** Ruiling Luan, Zhiling Mu, Fang Yue, Shaoying He

**Affiliations:** ^1^Department of Pharmacy, The Affiliated Yantai Yuhuangding Hospital of Qingdao University, Yantai, China; ^2^Department of Pediatrics, The Affiliated Yantai Yuhuangding Hospital of Qingdao University, Yantai, China

**Keywords:** attention deficit hyperactivity disorder, efficacy, tolerability, interventions, network meta-analysis

## Abstract

**Background:**

Our study is an analysis of multiple publications involving assessing the comparable efficacy and tolerability of six interventions, which are lisdexamfetamine dimesylate (LDX), atomoxetine (ATX), methylphenidate (MPH), clonidine hydrochloride (CLON), guanfacine extended release (GXR), and bupropion, for young patients (6–18 years old) suffering from attention deficit hyperactivity disorder (ADHD).

**Methods:**

A conventional meta-analysis (MA) was performed to give direct comparisons and a network meta-analysis (NMA) was used to show the combination of direct and indirect evidence. Ranking preference for all the interventions under a certain outcome was given by the surface of cumulative ranking curve area (SUCRA).

**Results:**

Overall, 15,025 participants from 73 studies were involved in our analysis. In the pairwise MA, LDX was associated with less withdrawal than ATX for lack of efficacy. MPH showed less effectiveness than LDX according to ADHD Rating Scale score. Based on the analysis of our NMA, significant results of efficacy that LDX is a competitive drug were observed when evaluating LDX in comparison with other drugs except for CLON. ATX and GXR presented higher rates of abdominal pain morbidity versus inactive treatment.

**Conclusion:**

The stimulants LDX and MPH are still highly recommended because they are highly effective and are tolerated well by patients. Among the non-stimulants, CLON can be taken into consideration for its appreciable effectiveness and tolerability. ATX and GXR can be seen as moderate choices.

## Introduction

Attention deficit hyperactivity disorder (ADHD) is a common kind of psychological behavior disorder that occurs in approximately 5% of children or adolescents (6–18 years of age) worldwide (1, 2). It is a relatively long-lasting disorder, the effects of which can be present for several years and in some cases even last an entire lifetime (2). Children or adolescents with ADHD are characterized by behaviors such as inattention, hyperactivity, and impulsivity (3). Such symptoms are usually detected during youth, between the ages of three and six (2). ADHD has increasingly attracted the attention of parents, doctors, and scientists because of its severely impact on daily activities in patients’ academic and social life (1). The exact causes of ADHD are unclear, but it is thought that it is the result of an imbalance of catecholamine metabolism in the cerebral cortex, or inhibitory dopaminergic and decrease of noradrenergic activities, or a mixture of the two (1, 4).

Drugs therapies treating ADHD can be classified into dopaminergic and noradrenergic pathways (4). Several drugs have been employed for patients suffering from ADHD, including stimulants and non-stimulants. For example, atomoxetine (ATX) is widely used and is considered as a non-psychostimulant (5). It can reduce the symptoms of ADHD and has other clinical advantages such as drowsiness, comorbidities with tics, and anxiety (6). Bupropion (BUP) is a monocyclic phenylaminoketone structurally related to the phenylisopropylamines, with significant antidepressant effects (7). Clonidine hydrochloride (CLON) is α_2_-adrenergic agonist developed to reduce and delay the release and has recently been used in combination with psychostimulants to treat ADHD (8, 9). Guanfacine is the most widely prescribed psychostimulants as the selective α_2A_-adrenoceptor agonist for the treatment of ADHD. The efficacy of guanfacine extended release (GXR) is considered to be among first-line treatments for ADHD (10). Lisdexamfetamine dimesylate (LDX) is a stimulant, normally used as a monotherapy or supplement for psychostimulants in ADHD treatment (10, 11). In general, LDX is employed as prodrug due to its fast absorption and hydroxylation, which leads to a gradual and long lasting release (12–14). Methylphenidate (MPH) is a psych stimulant and is considered as the first-line therapy (6). It can increase the concentration of serotonin, dopamine, and norepinephrine to control the extent of inattention and impulsivity (5). MPH is also recognized for its significant antidepressant effects (7). All of the treatments described above have been effective in the treatment of ADHD, but there are aspects of their relative efficacy and safety that still remain unclear. Therefore, it is necessary to establish a system of evaluation in order to make comparisons among these various therapies.

A variety of studies have been conducted in the attempt to create a system of comparison for these diverse treatments, however, a large proportion of these studies have only focused on the pairwise comparison with placebo-controlled treatment (7, 15–19), or were restricted to three or four medications (20). Besides, the majority of these current literatures for systematic analysis have only considered the efficacy of these therapies without incorporating an evaluation of their safety (21). Moreover, the characteristics of the samples chosen in some studies are limited to small subgroups and this has led to limitations in comparing the differences between treatments (4). More importantly, previous studies have used different indicators to measure the efficacy of treatment options. This has led to inconsistencies and contradictions that further increase the need to perform a network meta-analysis (NMA) to estimate the effectiveness and reliability of medications used to cure ADHD.

Our study tries to combine studies involving seven interventions (non-stimulants: ATX, BUP, CLON, and GXR; stimulants: LDX and MPH). Seven outcomes have been considered, including ADHD Rating Scale (ADHD-RS), all cause withdrawal, withdrawal due to adverse event, withdrawal due to lack of efficacy, nausea, abdominal pain, and fatigue.

## Materials and Methods

### Publication Search

A systematic search was conducted in PubMed, Embase, Cochrane library, and CNKI (up to March 29, 2017), aiming to retrieve randomized controlled trials (RCTs) related to drug therapy in children and juveniles with ADHD. We used the following key words: “randomized controlled trial,” “attention deficit hyperactivity disorder” (including synonyms), and “drug therapy.” The cited articles of references including RCTs, systematic reviews or meta-analyses were also searched manually as supplementary material. Ethical approval was not needed for this study.

### Inclusion Criteria

*Type of study*: mostly RCTs of a minimum of 3-week duration will be included in this review.

*Type of participants*: children and adolescents aged between 6 and 18 who meet Diagnostic and Statistical Manual of Mental Disorders, 4th edition (DSM-IV) criteria for ADHD.

*Type of interventions*: studies involving direct comparison with one drug therapy against another or against placebo will be included. All target interventions are ATX, CLON, GXR, BUP, LDX, and MPH.

*Type of outcome measures*: the efficacy is evaluated by ADHD-RS as a continuous score. And the adverse effects (as a dichotomous outcome) for tolerability are all cause withdrawals, withdraw due to adverse event, withdrawal due to lack of efficacy, nausea, abdominal pain, or fatigue for tolerability. Weight loss is not included because there is limited outcome on it.

### Data Extraction

We extracted baseline data and evaluated the risk of bias, including selection bias, performance bias, detection bias and other bias, by the means of random sequence generation, allocation concealment, blinding, incomplete outcome data, and selective reporting. The assessment tool came from Cochrane Handbook (version 5.1.0) for RCTs. Data of interest were blinding, durations, diagnostic criteria, treatment, age of patients, number of patients, and assessment criteria for patients’ conditions.

### Statistical Analysis

Conventional meta-analysis (MA) was performed by STATA 12.0 software, which gave us direct comparisons among these drugs, in the forms of the mean deviation (MD) with the corresponding 95% confidence interval (CI) for the primary outcomes of ADHD-RS, and the pooled odds ratios (ORs) with 95% CI for the secondary outcomes of tolerability. Cochran’s *Q* test (22) and the *I*^2^ test (23) were utilized to assess the degree of heterogeneity among studies. A fixed-effects model (Mantel-Haenszel method) was utilized if significant heterogeneity did not exist (*P* > 0.05 or *I*^2^ < 50%). Otherwise, a random-effects model was used.

This NMA was conducted using STATA 12.0 software and WinBUGS software, which showed us the combination of direct and indirect evidence. A Markov chain Monte Carlo method was applied to build Bayesian networks. The data presented in our NMA were similar to that in a pairwise MA. We illustrated the comparison of efficacy and tolerability by computing the MD and OR with the corresponding 95% credible interval (CrI) separately. Ranking preference for all the interventions under a certain outcome was given by the surface of the SUCRA, the value of which was 1 for the best and 0 for the worst.

There are five domains are bias due to (1) the randomization process, (2) deviations from intended interventions, (3) missing outcome data, (4) measurement of the outcome, and (5) selection of the reported results (24). We used a comparison adjusted funnel plot to illustrate publication bias. Symmetry of the plots indicated no publication bias. *P*-value was a significant parameter to assess whether there was consistency in comparing direct and indirect evidence, and *P* < 0.05 showed a statistical inconsistency. The degree of consistency was illustrated by color in the heat plot.

## Results

### Characteristics of Trials and Patients

**Figure 1:**
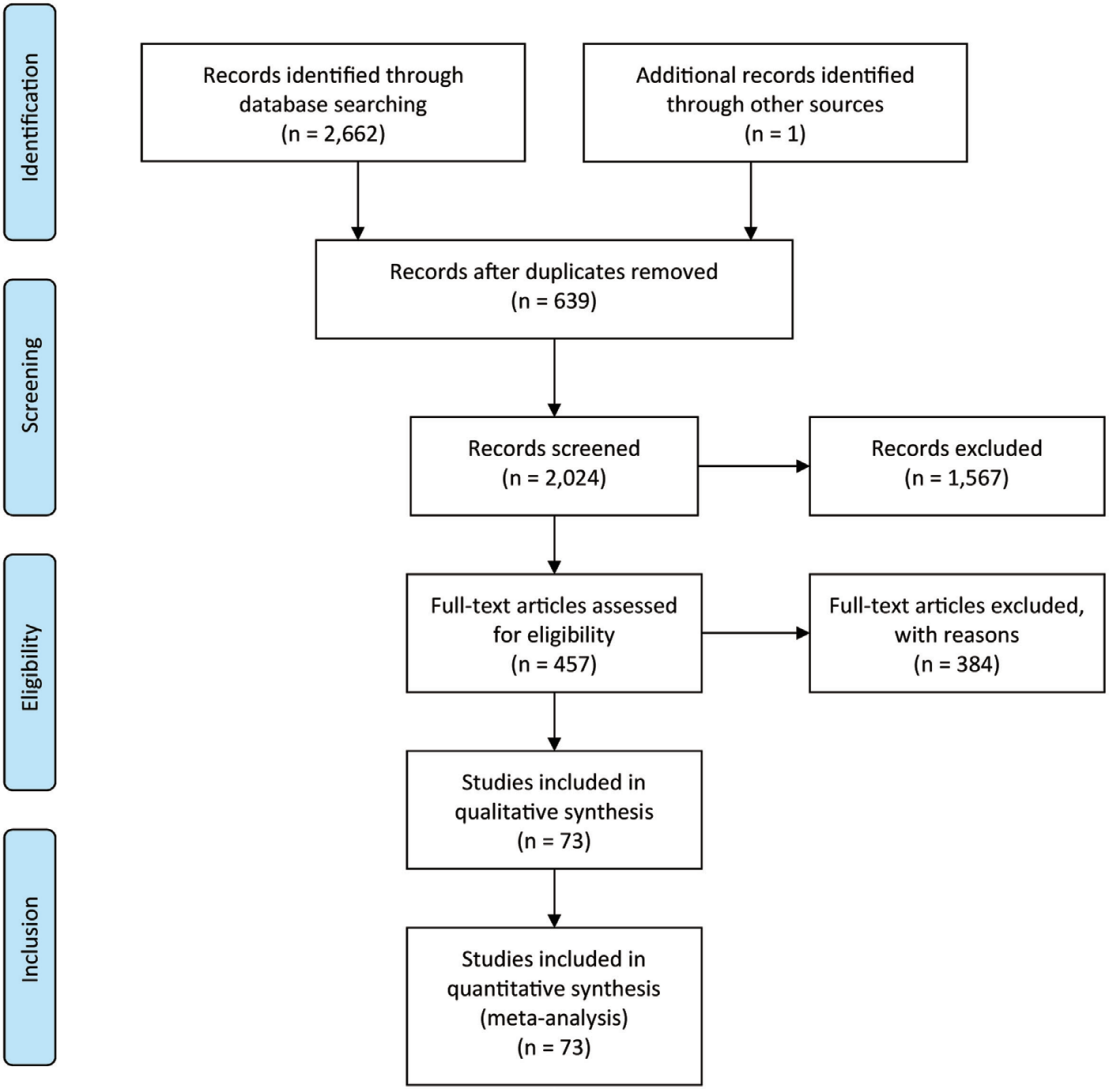
PRISMA flow chart.

**Figure 2:**
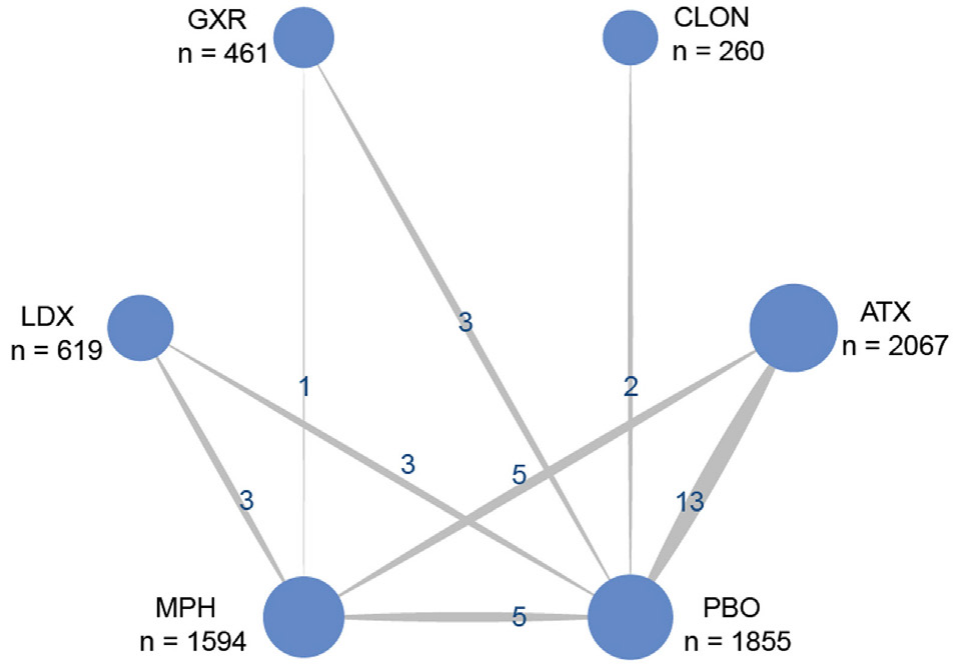
Network of randomized controlled trials (RCTs) comparing attention deficit hyperactivity disorder-rating scale (ADHD-RS) for attention deficit hyper activity disorder. The width of the lines is proportional to the number of trials comparing each pair of treatments and the numbers on the lines illustrate the exact number of trials included in the comparison; the area of the circles represents the cumulative number of patients for each intervention. PBO, placebo; ATX, atomoxetine; BUP, bupropion; CLON, clonidine hydrochloride; GXR, guanfacine extended release; LDX, lisdexamfetamine dimesylate; MPH, methylphenidate.

**Figure 3:**
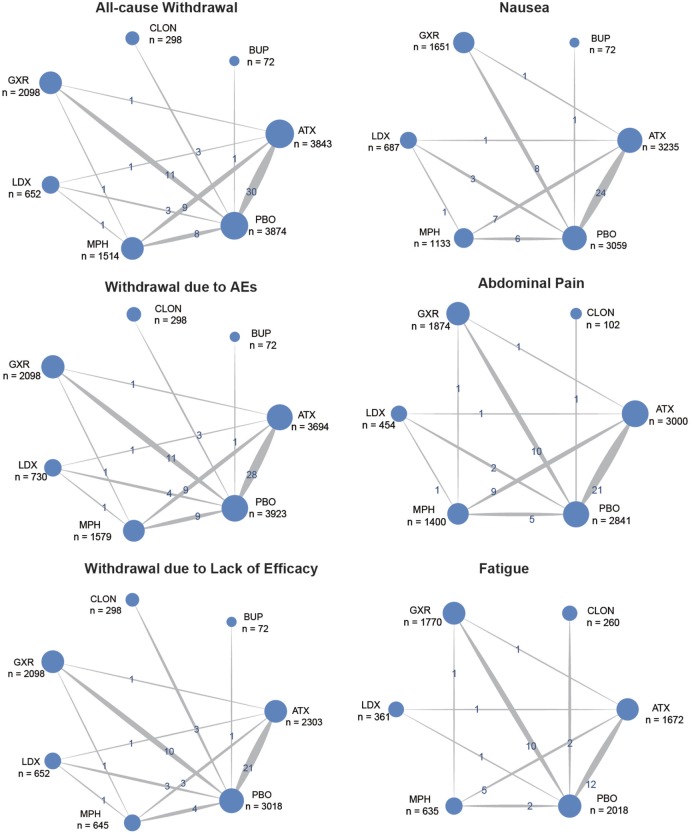
Network of randomized controlled trials (RCTs) comparing secondary outcomes of different treatments for attention deficit hyperactivity disorder. The width of the lines is proportional to the number of trials comparing each pair of treatments and the numbers on the lines illustrate the exact number; the area of the circles represents the cumulative number of patients for each intervention. PBO, placebo; ATX, atomoxetine; BUP, bupropion; CLON, clonidine hydrochloride; GXR, guanfacine extended release; LDX, lisdexamfetamine dimesylate; MPH, methylphenidate.

Figure 1 showed the process of literature retrieval and screening. A total of 2,024 studies were obtained after removing duplicates in the primary searches in databases and other sources. Among these studies, 1,567 trials were excluded by title and abstract, according to the inclusion criteria. Full-text reading finally enabled us to identify 73 qualitative trials as sources for data extraction (3, 5–16, 19, 25–83). Overall, 15,025 participants were involved in our analysis. Figures 2 and 3 showed the geometric distribution of RCTs for the included outcomes, which were related to different aspects in efficacy and tolerability. Placebo was taken as the control group in most RCTs. There were considerable quantities of patients in the research results for ATX, MPH, LDX, and GXR. However, the number of patients with BUP and CLON as interventions was limited. Baseline characteristics were listed in Table 1. Double blinding was adopted in most trials, with one single blinding and seven open-label trials.

**Table 1:** Characteristics of included studies.

Reference	Country	NCT no.	RCT	Blinding	Criteria	Age	Male	*N*	Comparison	Outcomes						
										①	②	③	④	⑤	⑥	⑦
Newcorn et al. (82)	USA	NCT01081145	√	Double	DSM-IV-TR	10.7	234	315	GXR vs. PBO	√	√	√	√	√		
Su et al. (83)	China	NCT01065259	√	Open-label	DSM-IV	9.5	197	237	MPH vs. ATX		√	√	√		√	
McCracken et al. (81)	USA	NCT00429273	√	Double	DSM-IV	10.1	91	137	GXR vs. MPH	√	√	√	√		√	√
Wigal et al. (80)	USA	NCT01239030	√	Double	DSM-IV	10.5	154	230	MPH vs. PBO					√	√	
Wilens et al. (19)	USA	NCT01081132	√	Double	DSM-IV-TR	14.5	202	312	GXR vs. PBO		√	√	√	√	√	√
Handen et al. (77)	USA	NCT00844753	√	Double	DSM-IV-TR	8.6	51	64	ATX vs. PBO		√	√	√			
Wehmeier et al. (79)	Germany	NCT00546910	√	Double	DSM-IV-TR	9.1	97	125	ATX vs. PBO		√	√	√			
Abikoff (42)	USA	NA	√	Double	DSM-IV	NA	85	114	MPH vs. PBO	√							
Shang et al. (6)	Taiwan	NCT00916786	√	Open-label	DSM-IV	9.90	140	160	ATX vs. MPH	√	√	√		√	√	
Setyawan et al. (78)	USA	NCT00763971	√	Double	DSM-IV-TR	10.8	167	211	LDX vs. MPH	√						
Hervas et al. (74)	USA	NCT01244490	√	Double	NA	10.9	163	338	GXR vs. ATX	√	√	√	√	√	√	√
Findling et al. (16)	USA	NA	√	Double	DSM-IV	10.8	188	272	GXR vs. PBO						√	√
Cutler et al. (72)	USA and UK	NCT00734578	√	Double	DSM-IV	10.8	326	455	GXR vs. PBO		√	√	√	√	√	√
Rugino (76)	USA	NCT01156051	√	Double	NA	9.15	17	29	GXR vs. PBO	√					√	√
Coghill et al. (13, 14)	USA	NCT00784654	√	Double	DSM-IV-TR	11	123	157	LDX vs. PBO	√		√				
Garg et al. (73)	India	NA	√	Double	NA	8.47	56	69	MPH vs. ATX		√	√		√	√	√
Lin et al. (75)	USA	NCT00922636	√	Double	DSM-IV-TR	11.4	80	89	PBO vs. MPH		√	√	√	√		√
Coghill et al. (13, 14)	UK	NA	√	Double	NA	10.9	178	222	LDX vs. MPH	√						
Coghill et al. (15)	EUROPE	NCT00763971	√	Double	DSM-IV	10.9	177	336	LDX vs. MPH	√	√	√	√	√	√	
Newcorn et al. (69)	USA	NCT00997984	√	Double	DSM-IV	9.1	235	333	GXR vs. PBO	√	√	√	√	√	√	√
Wietecha et al. (71)	USA	NCT00607919	√	Double	DSM-IV	NA	NA	209	PBO vs. ATX		√	√	√	√		√
Simonoff et al. (70)	UK	ISRCTN68384912	√	Double	NA	11.2	85	122	PBO vs. MPH		√	√	√			
Findling et al. (68)	USA	NCT00764868	√	Double	DSM-IV-TR	14.6	187	269	LDX vs. PBO		√	√	√			
Dittmann et al. (12)	EUROPE	NCT01106430	√	Double	DSM-IV-TR	10.9	197	262	LDX vs. ATX		√	√	√	√	√	√
Wilens et al. (10)	USA	NCT00734578	√	Double	DSM-IV	11.0	320	454	GXR vs. GXR		√	√	√	√	√	√
Harfterkamp et al. (3)	Netherland	NCT00380692	√	Double	DSM-IV	9.9	83	97	ATX vs. PBO	√	√	√	√	√	√	√
Yang et al. (67)	China	NCT01065259	√	Single	DSM-IV	9.5	119	262	MPH vs. ATX		√	√	√			
Dittmann et al. (62)	Germany	NA	√	Double	DSM-IV	10.9	NA	180	ATX vs. PBO		√	√	√	√		√
Kratochvil et al. (65)	USA	NCT00561340	√	Double	DSM-IV	6.1	63	88	ATX vs. PBO	√	√	√	√			
Findling et al. (63)	USA	NCT00735371	√	Double	DSM-IV	14.6	219	312	LDX vs. PBO					√		√
Wehmeier et al. (66)	Germany	NCT00546910	√	Double	DSM-IV	9.1	97	125	ATX vs. PBO		√	√	√	√	√	√
Kollins et al. (9, 64)	USA	NA	√	Double	DSM-IV	12.6	124	178	GXR vs. PBO	√	√	√	√		√	
Jain et al. (8)	USA	NCT00556959	√	Double	DSM-IV	9.6	165	236	CLON vs. PBO	√	√	√	√			√
Kollins et al. (9, 64)	USA	NCT00641329	√	Double	DSM-IV	10.4	145	198	CLON vs. PBO	√	√	√	√		√	√
Yildiz et al. (5)	Turkey	NA	√	Open-label	DSM-IV	9.8	22	25	ATX vs. MPH					√	√	
Martenyi et al. (60)	USA	NCT00386581	√	Double	DSM-IV	9.9	90	105	ATX vs. PBO	√	√	√		√	√	
Thurstone et al. (61)	USA	NCT00399763	√	Double	DSM-IV	16.06	55	70	ATX vs. PBO		√	√	√	√	√	
Connor et al. (59)	USA	NCT00367835	√	Double	DSM-IV	9.4	147	217	GXR vs. PBO		√	√	√	√	√	√
Dell’Agnello et al. (54)	Italy	NA	√	Double	DSM-IV	9.7	127	137	ATX vs. PBO		√	√		√	√	
Bedard (31)	Canada	NA	√	Double	DSM-IV	NA	100	118	MPH vs. PBO	√		
Svanborg et al. (56, 57)	Sweden	NA	√	Double	DSM-IV	11.6	NA	99	ATX vs. PBO	√	√			√	√	√
Takahashi et al. (58)	Japan	NA	√	Double	DSM-IV	10.25	209	245	ATX vs. PBO	√	√	√	√	√	√	
Block et al. (52)	USA	NCT00486122	√	Double	DSM-IV	8.8	209	288	ATX vs. PBO		√	√	√	√	√	
De Jong et al. (53)	Netherland	NCT00191906	√	Double	DSM-IV	10	NA	76	ATX vs. PBO		√	√	√			
Svanborg et al. (56, 57)	Sweden	NA	√	Double	DSM-IV	11.5	80	99	ATX vs. PBO		√	√	√			
Sallee et al. (55)	USA	NCT00150618	√	Double	DSM-IV	11	233	324	GXR vs. PBO		√	√	√			
Bangs et al. (49)	USA	NA	√	Double	DSM-IV	9.5	211	226	ATX vs. PBO		√	√	√	√		√
Newcorn et al. (51)	USA	NA	√	Double	DSM-IV	10.3	328	516	ATX vs. MPH	√	√	√		√	√	√
Biederman et al. (11)	USA	NA	√	Double	DSM-IV	10.6	257	345	GXR vs. PBO		√	√	√	√	√	√
Findling et al. (50)	USA	MCT00444574	√	Double	DSM-IV	8.8	127	176	MPH vs. PBO		√	√		√		
Biederman et al. (44)	USA	NA	√	Double	DSM-IV	9.0	201	285	LDX vs. PBO	√	√	√	√	√	√	
Gau et al. (46)	Taiwan	NA	√	Double	DSM-IV	9.1	94	106	ATX vs. PBO		√	√	√	√	√	
Atomoxetine ADHD and Comorbid MDD Study Group et al. (43)	USA	NA	√	Double	DSM-IV	14.6	104	142	ATX vs. PBO		√	√	√	√	√	√
Geller et al. (47)	USA	NA	√	Double	DSM-IV	12.2	114	176	ATX vs. PBO		√	√	√	√	√	
Wang et al. (48)	USA	NA	√	Double	DSM-IV	9.4	270	330	ATX vs. MPH	√	√	√		√	√	√
Buitelaar et al. (45)	Netherland	NA	√	Double	DSM-IV	10.7	146	158	ATX vs. PBO	√						√
Wilens et al. (41)	USA	NA	√	Double	DSM-IV	14.8	142	177	MPH vs. PBO	√	√	√	√	√	√	
Sumner et al. (40)	USA	NA	√	Double	DSM-IV	10.22	61	87	ATX vs. PBO		√	√	√	√	√	
Sangal et al. (39)	USA	NA	√	Double	DSM-IV	10.1	85	85	ATX vs. MPH		√	√	√		√	√
Weiss et al. (38)	Canada	NA	√	Double	DSM-IV	9.9	123	153	ATX vs. PBO	√	√	√				
Allen et al. (36)	USA	NA	√	Double	DSM-IV	10.9	131	145	ATX vs. PBO		√	√	√	√	√	√
Kemner et al. (37)	USA	NA	NA	Open-label	DSM-IV-TR	8.7	489	517	MPH vs. ATX	√						
Kaplan et al. (34)	USA	NA	√	Double	DSM-IV	9.8	78	98	ATX vs. PBO	√	√	√	√	√	√	
Kelsey et al. (35)	USA	NA	√	Double	DSM-IV	9.5	139	197	ATX vs. PBO	√	√	√		√	√	
Biederman et al. (32)	USA	NA	√	Double	DSM-IV	9.06	104	136	MPH vs. PBO	√		√				
Hazell and Stuart (33)	Australia	NA	√	Double	DSM-IV	9.86	61	67	CLON vs. PBO		√	√	√			
Michelson et al. (29)	USA	NA	√	Double	DSM-IV	10.1	120	170	ATX vs. PBO	√	√	√		√	√	
Spencer et al. (30)	USA	NA	√	Double	DSM-IV	9.7	201	253	ATX vs. PBO	√	√	√		√	√	
Greenhill et al. (27)	USA	NA	√	Double	DSM-IV	9	257	314	MPH vs. PBO	√	√	√			√	
Kratochvil et al. (28)	USA	NA	√	Open-label	DSM-IV	10.4	211	228	ATX vs. MPH	√	√	√		√	√	
Michelson et al. (25)	USA	NA	√	Double	DSM-IV	11.3	212	297	ATX vs. PBO	√	√	√	√	√	√	
Scahill et al. (26)	USA	NA	√	Double	DSM-IV	10.4	NA	34	GXR vs. PBO	√						√
Conners et al. (7)	USA	NA	√	Double	DSM-III	8.5	NA	109	BUP vs. PBO		√	√	√	√		

*NA, not available; PBO, placebo; ATX, atomoxetine; BUP, bupropion; CLON, clonidine hydrochloride; GXR, guanfacine extended release; LDX, lisdexamfetamine dimesylate; MPH, methylphenidate; ①, attention deficit hyper activity disorder rating scale; ②, all-cause withdrawal; ③, withdrawal due to adverse events; ④, withdrawal due to lack of efficacy; ⑤, nausea; ⑥, abdominal pain; ⑦, fatigue*.

### Result from Pairwise MA

The results of the direct MA were shown in Table 2. All the included therapies had comparisons with placebo. In comparison to placebo, ATX had significantly better ADHD-RS. (MD = 6.95, 95% CI: 4.92–8.98) and decreasing withdrawal due to lack of efficacy (OR = 0.55, 95% CI: 0.40–0.74), but an increase the chance of adverse events (nausea: OR = 2.22, 95% CI: 1.61–3.03; abdominal pain: OR = 1.47, 95% CI: 1.16–1.85; fatigue: OR = 1.82, 95% CI: 1.33–2.50). There were no statistically significant results in the comparison of BUP versus placebo. CLON showed great improvement in the primary outcome of ADHD-RS (MD = 8.10, 95% CI: 4.47–11.73) and less withdrawal was observed (all-cause withdrawal: OR = 0.65, 95% CI: 0.43–0.97; withdrawal due to lack of efficacy: OR = 0.37, 95% CI: 0.20–0.66). Comparing GXR versus placebo, significant results were obtained concerning its ability to cause adverse effects (abdominal pain: OR = 2.04, 95% CI: 1.37–3.13; fatigue: OR = 2.70, 95% CI: 1.89–3.85) and related withdrawal due to adverse events (OR = 2.94, 95% CI: 1.41–5.88), but the chance of withdrawal due to lack of efficacy was reduced (OR = 0.41, 95% CI: 0.30–0.56). LDX significantly resulted in less withdrawal (all-cause withdrawal: OR = 0.67, 95% CI: 0.47–0.96; withdrawal due to lack of efficacy: OR = 0.18, 95% CI: 0.06–0.48) versus placebo. MPH showed a similar decrease in withdrawal (all-cause withdrawal: OR = 0.67, 95% CI: 0.50–0.91; withdrawal due to lack of efficacy: OR = 0.50, 95% CI: 0.33–0.77), plus an improvement in ADHD-RS (MD = 6.53, 95% CI: 4.91–8.15). In the direct comparisons among the seven interventions, LDX was associated with less withdrawal due to lack of efficacy (OR = 0.16, 95% CI: 0.04–0.73) than ATX and MPH showed less effectiveness than LDX according to ADHD-RS (MD = −5.80, 95% CI: −8.93 to −2.67).

**Table 2:** Relative treatment effects of direct meta-analyses.

Comparison	①	②	③	④	⑤	⑥	⑦
ATX vs. PBO	6.95 (4.92, 8.98)	0.95 (0.81, 1.11)	1.22 (0.83, 1.79)	0.55 (0.40, 0.74)	2.22 (1.61, 3.03)	1.47 (1.16, 1.85)	1.82 (1.33, 2.50)
BUP vs. PBO	–	2.04 (0.41, 10.00)	2.56 (0.29, 25.00)	1.54 (0.16, 14.29)	1.23 (0.40, 3.70)	–	–
CLON vs. PBO	8.10 (4.47, 11.73)	0.65 (0.43, 0.97)	1.45 (0.15, 14.29)	0.37 (0.20, 0.66)	–	1.41 (0.55, 3.57)	1.49 (0.26, 8.33)
GXR vs. PBO	5.56 (−2.84, 13.96)	0.90 (0.77, 1.05)	2.94 (1.41, 5.88)	0.41 (0.30, 0.56)	1.27 (0.88, 1.85)	2.04 (1.37, 3.13)	2.70 (1.89, 3.85)
LDX vs. PBO	7.45 (−0.31, 15.21)	0.67 (0.47, 0.96)	1.10 (0.38, 3.23)	0.18 (0.06, 0.48)	1.18 (0.23, 5.88)	0.51 (0.18, 1.41)	1.69 (0.36, 7.69)
MPH vs. PBO	6.53 (4.91, 8.15)	0.67 (0.50, 0.91)	1.35 (0.66, 2.78)	0.50 (0.33, 0.77)	1.41 (0.77, 2.56)	1.54 (0.87, 2.70)	2.56 (0.72, 9.09)
GXR vs. ATX	–	1.02 (0.54, 1.89)	1.75 (0.57, 5.26)	0.97 (0.27, 3.45)	0.58 (0.31, 1.11)	0.75 (0.33, 1.67)	1.18 (0.65, 2.13)
LDX vs. ATX	–	1.04 (0.61, 1.79)	0.84 (0.32, 2.17)	0.16 (0.04, 0.73)	0.80 (0.40, 1.59)	–	0.90 (0.40, 2.00)
MPH vs. ATX	0.94 (−0.34, 2.21)	0.81 (0.61, 1.05)	0.74 (0.48, 1.14)	0.40 (0.12, 1.37)	0.57 (0.37, 0.88)	0.75 (0.53, 1.05)	0.34 (0.14, 0.83)
MPH vs. GXR	−0.22 (−4.32, 3.88)	0.98 (0.35, 2.78)	1.96 (0.17, 20.00)	0.49 (0.04, 5.56)	0.67 (0.26, 1.69)	0.93 (0.45, 1.92)	0.22 (0.08, 0.63)
MPH vs. LDX	−5.80 (−8.93, −2.67)	1.16 (0.68, 2.00)	0.4 (0.08, 2.13)	2.00 (0.93, 4.35)	–	0.67 (0.18, 2.44)	–

*PBO, placebo; ATX, atomoxetine; BUP, bupropion; CLON, clonidine hydrochloride; GXR, guanfacine extended release; LDX, lisdexamfetamine dimesylate; MPH, methylphenidate; ①, attention deficit hyper activity disorder rating scale; ②, all-cause withdrawal; ③, withdrawal due to adverse events; ④, withdrawal due to lack of efficacy; ⑤, nausea; ⑥, abdominal pain; ⑦, fatigue*.

### Results from NMA

**Figure 4:**
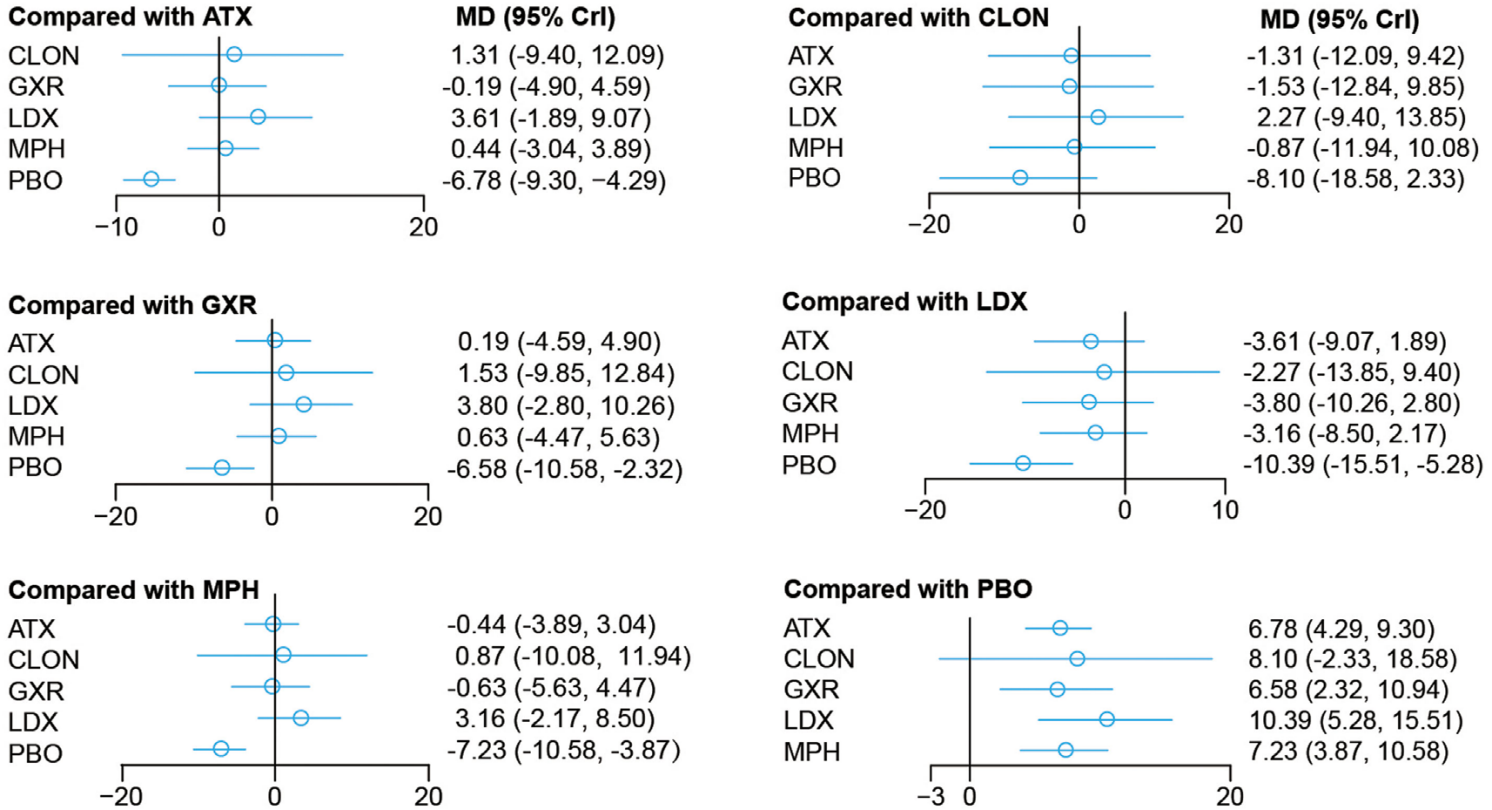
Odds ratios (95% credential intervals) for network comparison of ADHD-RS for attention deficit hyperactivity disorder. PBO, placebo; ATX, atomoxetine; BUP, bupropion; CLON, clonidine hydrochloride; GXR, guanfacine extended release; LDX, lisdexamfetamine dimesylate; MPH, methylphenidate.

**Figure 5:**
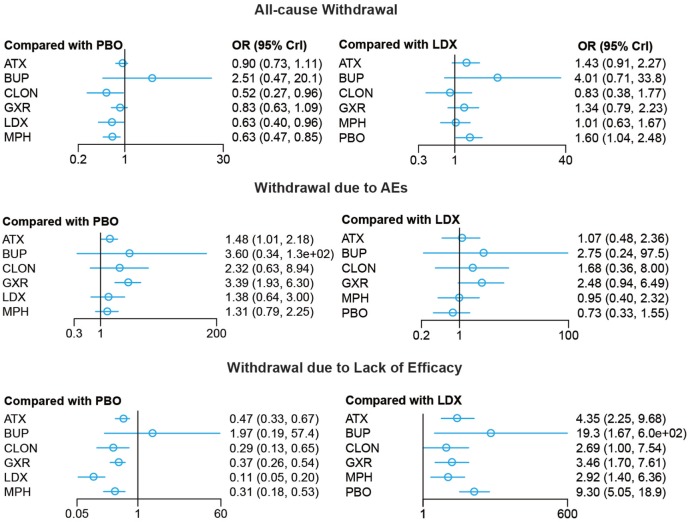
Odds ratios (95% credential intervals) for network comparison of withdrawal outcomes of different treatments for attention deficit hyperactivity disorder. PBO, placebo; ATX, atomoxetine; BUP, bupropion; CLON, clonidine hydrochloride; GXR, guanfacine extended release; LDX, lisdexamfetamine dimesylate; MPH, methylphenidate.

**Figure 6:**
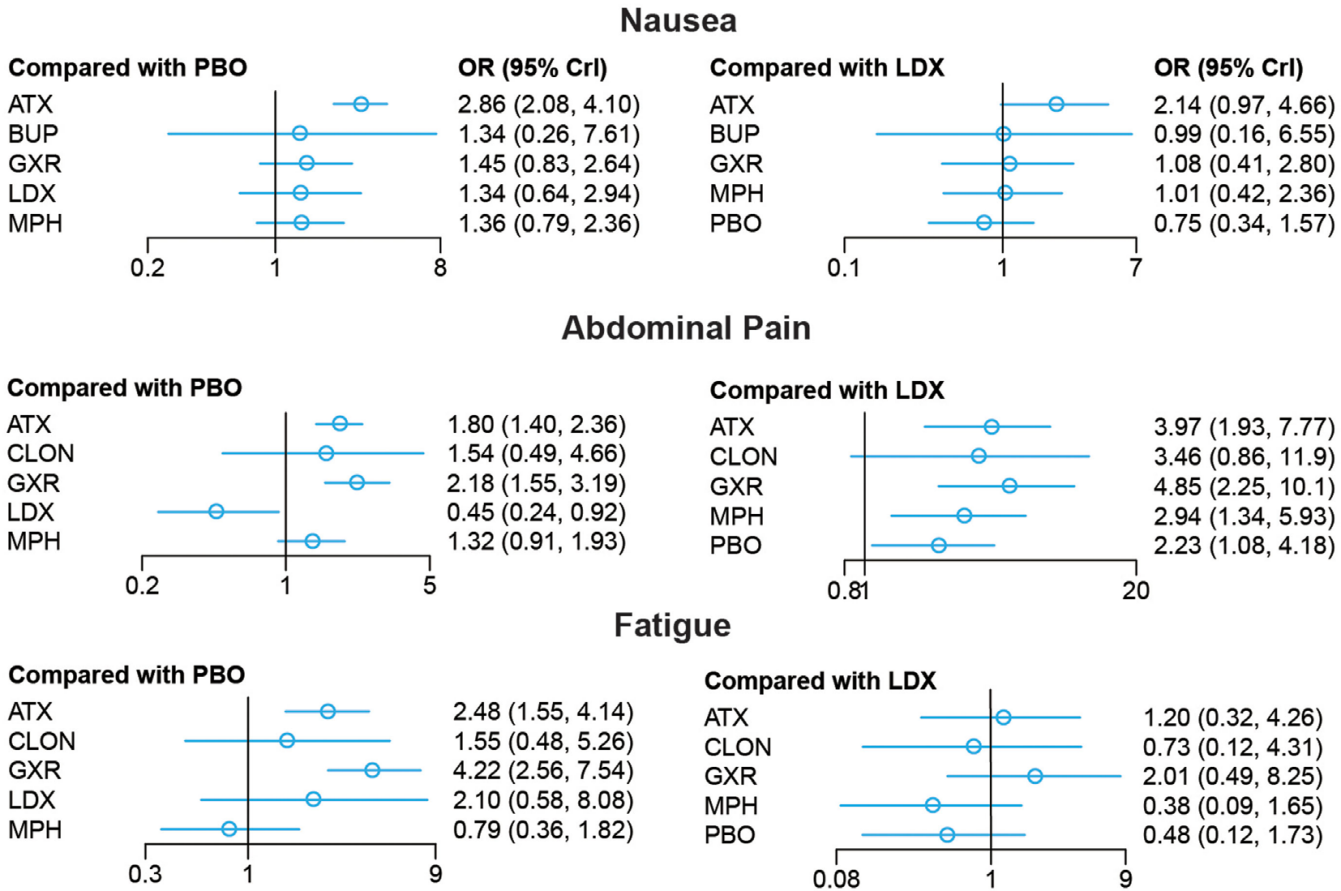
Odds ratios (95% credential intervals) for network comparison of adverse events of different treatments for attention deficit hyperactivity disorder. PBO, placebo; ATX, atomoxetine; BUP, bupropion; CLON, clonidine hydrochloride; GXR, guanfacine extended release; LDX, lisdexamfetamine dimesylate; MPH, methylphenidate.

Bayesian models allowed for more refined estimates. Comparisons without direct connection were compared indirectly through Bayesian NMA. Available data from NMA was recorded in Tables 3 and 4 and the results were graphically presented in the forest plots in Figures 4–6. For ADHD-RS, statistically significant improvement was obtained in comparisons with placebo (ATX: MD = 6.78, 95% CrI: 4.29–9.30; GXR: MD = 6.58, 95% CrI: 2.32–10.94; LDX: MD = 10.39, 95% CrI: 5.28–15.51; MPH: MD = 7.23, 95% CrI: 3.87–10.58). In terms of all-cause withdrawal, a significant decrease was observed in CLON, LDX and MPH versus placebo (CLON: OR = 0.52, 95% CrI: 0.27–0.96; LDX: OR = 0.63, 95% CrI: 0.40–0.96; MPH: OR = 0.63, 95% CrI: 0.47–0.85). For withdrawal due to adverse events, ATX and GXR showed a higher possibility versus placebo (ATX: OR = 1.48, 95% CrI: 1.01–2.18; GXR: OR = 3.39, 95% CrI: 1.93–6.30). GXR showed more association with adverse response which had led to withdrawal than ATX (OR = 2.29, 95% CrI: 1.20–4.57), and MPH presented reduction versus GXR (OR = 0.39, 95% CrI: 0.18–0.83). When it came to withdrawal caused by lack of efficacy, all interventions except for BUP presented greater effectiveness than placebo (ATX: OR = 0.47, 95% CrI: 0.33–0.67; CLON: OR = 0.29, 95% CrI: 0.13–0.65; GXR: OR = 0.37, 95% CrI: 0.26–0.54; LDX: OR = 0.11, 95% CrI: 0.05–0.20; MPH: OR = 0.31, 95% CrI: 0.18–0.53). Significant results were acquired when evaluating LDX with other drugs except for CLON (ATX: OR = 0.23, 95% CrI: 0.10–0.44; BUP: OR = 0.05, 95% CrI: 0.01–0.60; GXR: OR = 0.29, 95% CrI: 0.13–0.59; MPH: OR = 0.34, 95% CrI: 0.16–0.72), which indicated the considerable clinical performance of LDX as the participants expected.

**Table 3:** Network meta-analysis results of ADHD rating scale.

**ATX**	1.31 (−9.42, 12.09)	−0.19 (−4.90, 4.59)	3.61 (−1.89, 9.07)	0.44 (−3.04, 3.89)	−6.78 (−9.30, −4.29)
−1.31 (−12.09, 9.42)	**CLON**	−1.53 (−12.84, 9.85)	2.27 (−9.40, 13.85)	−0.87 (−11.94, 10.08)	−8.10 (−18.58, 2.33)
0.19 (−4.59, 4.90)	1.53 (−9.85, 12.84)	**GXR**	3.80 (−2.80, 10.26)	0.63 (−4.47, 5.63)	−**6.58 (**−**10.94**, −**2.32)**
−3.61 (−9.07, 1.89)	−2.27 (−13.85, 9.40)	−3.80 (−10.26, 2.80)	**LDX**	−3.16 (−8.50, 2.17)	−**10.39 (**−**15.51**, −**5.28)**
−0.44 (−3.89, 3.04)	0.87 (−10.08, 11.94)	−0.63 (−5.63, 4.47)	3.16 (−2.17, 8.50)	**MPH**	−**7.23 (**−**10.58**, −**3.87)**
**6.78 (4.29, 9.30)**	8.10 (−2.33, 18.58)	**6.58 (2.32, 10.94)**	**10.39 (5.28, 15.51)**	**7.23 (3.87, 10.58)**	**PBO**

*PBO, placebo; ATX, atomoxetine; BUP, bupropion; CLON, clonidine hydrochloride; GXR, guanfacine extended release; LDX, lisdexamfetamine dimesylate; MPH, methylphenidate*.

*The boldface values mean statistical significance*.

**Table 4:** Network meta-analysis results of withdrawals and adverse events for ADHD disorder.

		ATX	BUP	CLON	GXR	LDX	MPH	PBO	
All-cause withdrawal	ATX	1	0.46 (0.09, 2.72)		**0.51 (0.26, 0.96)**	0.47 (0.21, 1.03)	**0.47 (0.28, 0.79)**	**0.35 (0.24, 0.48)**	Nausea
	BUP	2.80 (0.51, 22.65)	1		1.09 (0.18, 6.30)	1.01 (0.15, 6.23)	1.02 (0.17, 5.81)	0.75 (0.13, 3.86)	
	CLON	0.58 (0.29, 1.09)	0.20 (0.02, 1.22)	1					
	GXR	0.92 (0.66, 1.28)	0.33 (0.04, 1.82)	1.60 (0.82, 3.22)	1	0.92 (0.36, 2.41)	0.93 (0.42, 2.05)	0.69 (0.38, 1.21)	
	LDX	0.70 (0.44, 1.09)	0.25 (0.03, 1.40)	1.21 (0.57, 2.61)	0.75 (0.45, 1.26)	1	1.01 (0.42, 2.36)	0.75 (0.34, 1.57)	
	MPH	0.70 (0.53, 0.94)	0.25 (0.03, 1.38)	1.22 (0.63, 2.48)	0.76 (0.52, 1.13)	1.01 (0.63, 1.67)	1	0.73 (0.42, 1.27)	
	PBO	1.12 (0.90, 1.36)	0.40 (0.05, 2.14)	**1.93 (1.05, 3.67)**	1.21 (0.91, 1.58)	**1.60 (1.04, 2.48)**	**1.58 (1.17, 2.12)**	1	
Withdrawal due to adverse events	ATX	1		0.86 (0.26, 2.64)	1.22 (0.82, 1.86)	**0.25 (0.13, 0.52)**	0.73 (0.52, 1.04)	**0.55 (0.42, 0.71)**	Abdominal pain
	BUP	2.44 (0.22, 87.36)	1						
	CLON	1.57 (0.40, 6.30)	0.63 (0.02, 9.68)	1	1.42 (0.45, 4.76)	0.29 (0.08, 1.16)	0.85 (0.27, 2.86)	0.65 (0.21, 2.03)	
	GXR	**2.29 (1.20, 4.57)**	0.94 (0.03, 10.91)	1.46 (0.34, 6.23)	1	**0.21 (0.10, 0.44)**	**0.61 (0.37, 0.95)**	**0.46 (0.31, 0.64)**	
	LDX	0.93 (0.42, 2.10)	0.38 (0.01, 4.57)	0.59 (0.12, 2.77)	0.40 (0.15, 1.06)	1	**2.94 (1.34, 5.93)**	**2.23 (1.08, 4.18)**	
	MPH	0.89 (0.55, 1.46)	0.36 (0.01, 4.10)	0.57 (0.14, 2.39)	**0.39 (0.18, 0.83)**	0.95 (0.40, 2.32)	1	0.76 (0.52, 1.09)	
	PBO	**0.68 (0.46, 0.99)**	0.28 (0.01, 2.92)	0.43 (0.11, 1.6)	**0.30 (0.16, 0.52)**	0.73 (0.33, 1.55)	0.76 (0.44, 1.26)	1	
Withdrawal due to lack of efficacy	ATX	1		0.62 (0.17, 2.27)	1.68 (0.89, 3.39)	0.84 (0.23, 3.1)	**0.32 (0.15, 0.68)**	**0.40 (0.24, 0.64)**	Fatigue
	BUP	4.26 (0.41, 127.74)	1						
	CLON	0.61 (0.26, 1.51)	0.15 (0.01, 1.67)	1	2.75 (0.75, 10.38)	1.36 (0.23, 8.08)	0.51 (0.12, 2.23)	0.64 (0.19, 2.10)	
	GXR	0.79 (0.49, 1.30)	0.19 (0.01, 1.99)	1.30 (0.52, 3.06)	1	0.50 (0.12, 2.01)	**0.19 (0.08, 0.45)**	**0.24 (0.13, 0.39)**	
	LDX	**0.23 (0.1, 0.44)**	**0.05 (0.01, 0.60)**	0.37 (0.13, 1.00)	**0.29 (0.13, 0.59)**	1	0.38 (0.09, 1.65)	0.48 (0.12, 1.73)	
	MPH	0.67 (0.36, 1.22)	0.16 (0.01, 1.68)	1.11 (0.42, 2.75)	0.84 (0.45, 1.60)	**2.92 (1.40, 6.36)**	1	1.26 (0.55, 2.77)	
	PBO	**2.14 (1.49, 3.03)**	0.51 (0.02, 5.16)	**3.49 (1.54, 7.54)**	**2.69 (1.86, 3.90)**	**9.30 (5.05, 18.92)**	**3.19 (1.88, 5.42)**	1	

*PBO, placebo; ATX, atomoxetine; BUP, bupropion; CLON, clonidine hydrochloride; GXR, guanfacine extended release; LDX, lisdexamfetamine dimesylate; MPH, methylphenidate*.

*Note that the lower half of a table is transposed, thus column treatments are compared against row treatments, vice versa*.

*The boldface values mean statistical significance*.

For adverse effects, including nausea, abdominal pain and fatigue, the results presented further reinforced initial estimates. There was less occurrence of nausea in the groups treated with GXR, MPH and placebo than those with ATX (GXR: OR = 0.51, 95% CrI: 0.26–0.96; MPH: OR = 0.47, 95% CrI: 0.28–0.79; placebo: 0.35, 95% CrI: 0.24–0.48). Statistically significant data was available in comparing morbidity of abdominal pain. A statistical decrease of morbidity was observed in LDX versus other drugs except for CLON (ATX: OR = 0.25, 95% CrI: 0.13–0.52; GXR: OR = 0.21, 95% CrI: 0.10–0.44; MPH: OR = 0.34, 95% CrI: 0.17–0.75; placebo: OR = 0.45, 95% CrI: 0.24–0.92). ATX and GXR presented higher morbidity of abdominal pain versus inactive treatment (ATX: OR = 1.80, 95% CrI: 1.40–2.36; GXR: OR = 2.18, 95% CrI: 1.55–3.19). MPH presented less abdominal pain than GXR (OR = 0.61, 95% CrI: 0.37–0.95). Similarly, ATX and GXR presented more fatigue than placebo (ATX: OR = 2.48, 95% CrI: 1.55–4.14; GXR: OR = 4.22, 95% CrI: 2.56–7.54) and MPH resulted in less fatigue than ATX and GXR (ATX: OR = 0.32, 95% CrI: 0.15–0.68; GXR: OR = 0.19, 95% CrI: 0.08–0.45).

### Ranking Scheme Based on SUCRA

**Figure 7:**
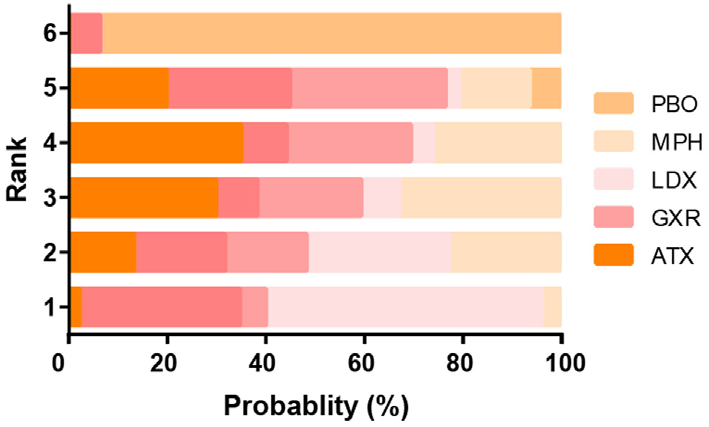
Ranking grams showing probability of each strategy having each specific rank (1–6) for attention deficit hyperactivity disorder-rating scale (ADHD-RS). Ranking indicates the quality of the individual treatment options, with one being the best and six being the worst. PBO, placebo; ATX, atomoxetine; BUP, bupropion; CLON, clonidine hydrochloride; GXR, guanfacine extended release; LDX, lisdexamfetamine dimesylate; MPH, methylphenidate.

**Figure 8:**
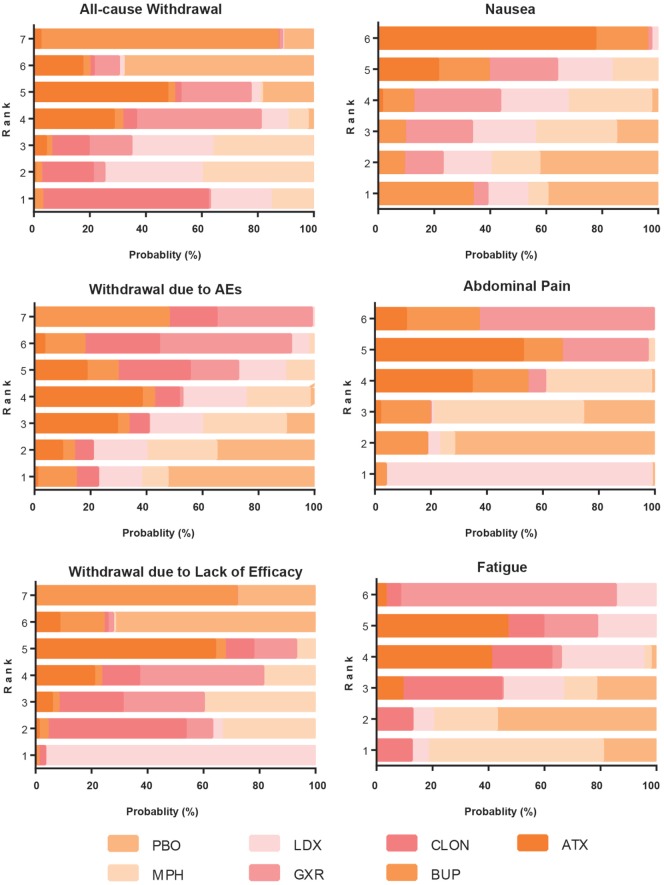
Ranking grams showing the probability of each strategy having each specific rank (1–7) for secondary outcomes. Ranking indicates the quality of the individual treatment options, with one being the best and seven being the worst. PBO, placebo; ATX, atomoxetine; BUP, bupropion; CLON, clonidine hydrochloride; GXR, guanfacine extended release; LDX, lisdexamfetamine dimesylate; MPH, methylphenidate.

The probability of being the best treatment was derived from SUCRA. The result was displayed in Figures 7 and 8. LDX and MPH could be considered as a group with the best comprehensive ranking score, including efficacy and tolerability. LDX had the highest probability of being the best efficacious drug therapy in decreasing ADHD symptoms (0.72) and remitting abdominal pain (0.82). MPH was located in the top three under all the outcomes except nausea. CLON ranked in the secondary group, but there was an absence of data related to nausea and abdominal pain. ATX and GXR had moderate rankings in efficacy, but were associated with the worst evaluation in the morbidity of adverse events. Though BUP was given a high ranking in reducing nausea, the results remained unclear due to the absence of essential information.

### Publication Bias and Consistency

The symmetry of the “comparison adjusted” funnel plots in Figure S1 in Supplementary Material suggested no publication bias. The small-study effect was limited in our research. According to the heat plot in Figure S2 in Supplementary Material, there were no statistically inconsistent results between direct and indirect evidence according to the blue color in most area, except for some ambiguity between LDX and MPH.

## Discussion

This comprehensive NMA was conducted to compare the efficacy and tolerability of all interventions used to treat young ADHD patients. Our NMA study gave a comprehensive evaluation that included direct and indirect evidence extracted from previous studies. This NMA’s results indicated that the statistical differences between all interventions were compared successfully and their ranking orders under different criteria were determined, respectively. Therefore, it is possible for us to find the optimal treatment option by taking all the outcomes into account.

Stimulants, including LDX and MPH, have been comprehensively proven superior to non-stimulants in improving children and adolescents’ symptoms of ADHD both in documents and our NMA study. LDX presented the best efficacy. Previous studies have demonstrated that the mechanism of LDX for treating ADHD was related to the sufficient gradual-release of d-amphetamine by hydrolysis from LDX (44). Due to this feature, LDX could provide continuous effects throughout the day. LDX is an effective medication, but its level of toxicity is generally attracted additional attention because it frequently results in adverse events. Nevertheless, it has been confirmed that its toxicity can be controlled *via* changing the daily dosage (44), which makes it very competitive and consistent with our NMA result.

Besides, our results suggested that MPH is a good candidate, especially with regards to fatigue and the rate of withdrawal, which makes MPHas the routine therapy clinically. LDX is used for patients with ADHD who have an inadequate response to MPH (12, 14). Although MPH releases quickly *in vivo* as a stimulant, it can be controlled by modification, which is documented as osmotic release oral system (37, 41, 51, 83), leading to moderate side effects (20). It is worth noting that the adverse responses that have been selected in our NMA are related to mild symptoms. There are still arguments concerning the stimulant therapies. For example, some researchers have mentioned that stimulants are not recommended for patients with various cardiovascular problems because there have been reports of sudden death at usual doses and serious cardiovascular adverse events (68).

Non-stimulant therapies can reduce safety concerns to some extent, so they are expected to be substitutes for stimulants. We give an appreciable ranking score to CLON, and its performance in ADHD-RS shows that it tends to be superior to the stimulant MPH. Although its characteristics of releasing in a twinkling induces unwanted effects, for instance, somnolence, restfulness, and sleepiness, and its half-life is relatively short, leading to more side effects, dose optimization and extended-release formulation could be adapted to address the problems (8). However, the probability of overestimation remains due to the relatively limited sample size and direct evidence is absent to provide robust support. ATX and GXR are located at moderate positions under symptom improvement and rate of withdrawal. There are not significant differences in ADHD-RS when compared with another therapy, which indicated comparable efficacy. This result is consistent with existed evidence. However, the unsatisfying SUCRA ranking scores of ATX and GXR in nausea, abdominal pain and fatigue do not mean they are poorly tolerated or unsafe. Statistics about BUP should be updated so that a more comprehensive estimate can be presented.

The overall quality of literatures included can be considered relatively high in terms of publication time, the authority of the journals and the characteristics of the studies. Besides, this NMA study compared all available medications for ADHD and assessed their efficacy and tolerability under various outcomes. However, there are some limitations existing for our study. (1) It is difficult for us to exclude publication bias completely, especially for ADHD-RS and all-cause withdrawal. This may be a result of small sample size and lack of direct comparison between some therapies, such as GXR versus CLON, or CLON versus BUP. (2) Most of the studies compared active treatment to placebo and only a handful of comparative effectiveness studies. (3) Differences in samples, such as mean age, gender ratio, and growth background also affect the accuracy of our NMA. Thus, in order to take all the limitations mentioned above into consideration, better-designed RCT studies should be performed.

In summary, according to the results obtained from our NMA, the stimulants LDX and MPH are still highly recommended because they are highly efficacious and well tolerated by patients. Among the non-stimulants, CLON should be taken into consideration for its appreciable effectiveness and tolerability. ATX and GXR can be seen as moderate choices. We failed to evaluate BUP because of the lack of enhanced evidence. Even though this study provides some guidance for the effectiveness and safety of various medications, clinicians still need to use their professional judgment in assessing the benefits with the efficacy and reliability profile of the therapy, as well as patients’ features and preferences in order to make final treatment decisions.

## Author Contributions

Conception or design of the work: RL and ZM. Data acquisition, analysis, and interpretation: FY. Drafting the work: RL and ZM. Revising article critically for important intellectual content: SH. Final approval of the version to be published: all authors.

## Conflict of Interest Statement

The authors declare that the research was conducted in the absence of any commercial or financial relationships that could be construed as a potential conflict of interest.

## Supplementary Material

Figure S1 Publication bias of all clinical outcomes. The “comparison adjusted” funnel plot evaluates the publication bias of all clinical outcomes with the standard error on the vertical axis.

Figure S2 Heat plot. Consistency can be checked by contrasting effect estimates from direct comparisons with indirect evidence of all clinical outcomes. In combination, we show heat colors corresponding to the change in agreement between direct and indirect estimate. The blue color means the two data possess good consistency while red color indicates inconsistency.
