# Supplementary figures and images for: Efficacy and Tolerability of Different Interventions in Children and Adolescents with Attention Deficit Hyperactivity Disorder
Source: Front Psychiatry. 2017 Nov 13;8:229. doi: 10.3389/fpsyt.2017.00229 (PMC5694170; doi:10.3389/fpsyt.2017.00229)

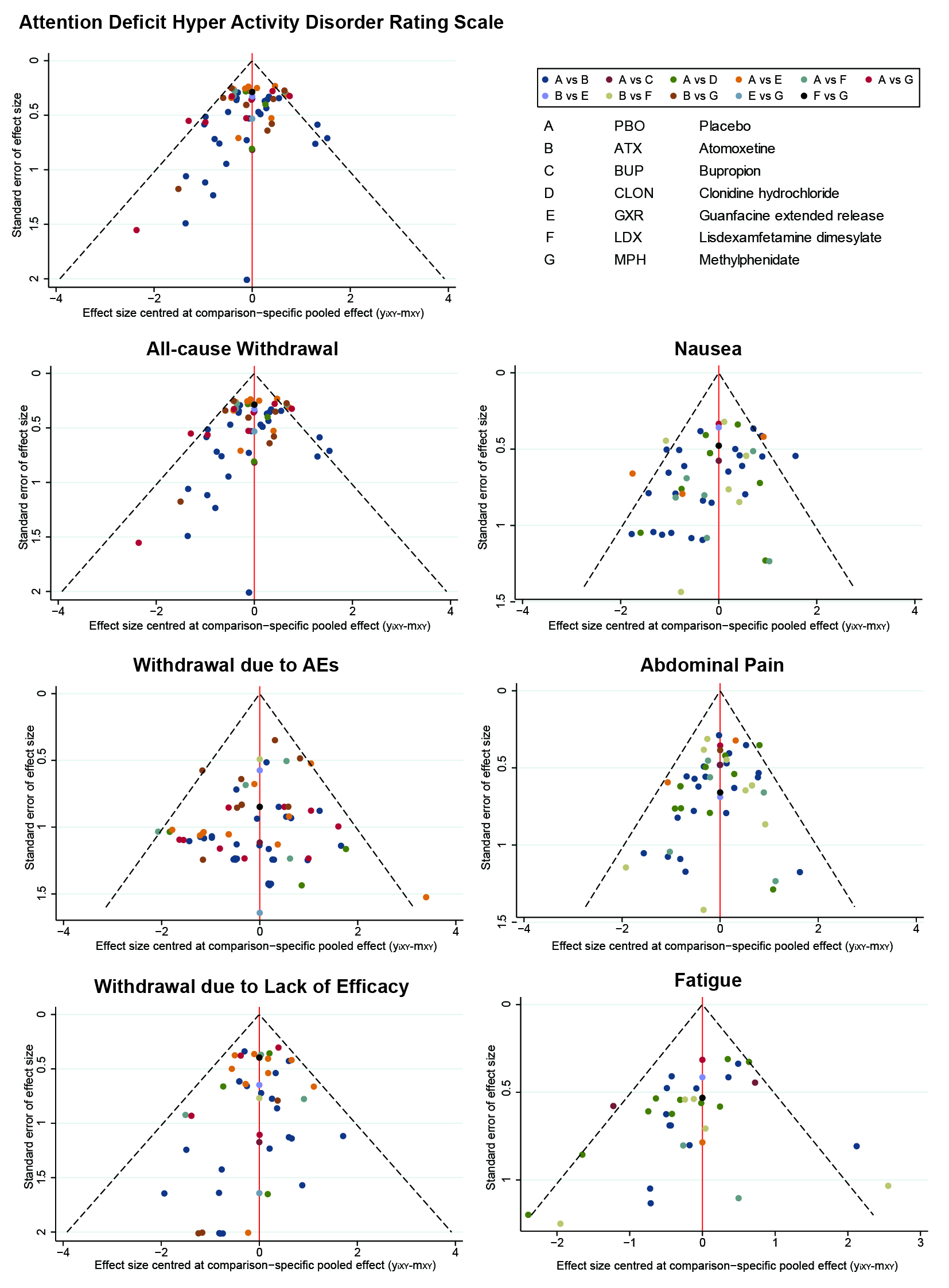

Supplement: Figure S1 — Publication bias of all clinical outcomes. The “comparison adjusted” funnel plot evaluates the publication bias of all clinical outcomes with the standard error on the vertical axis. [file Image_1.TIF]

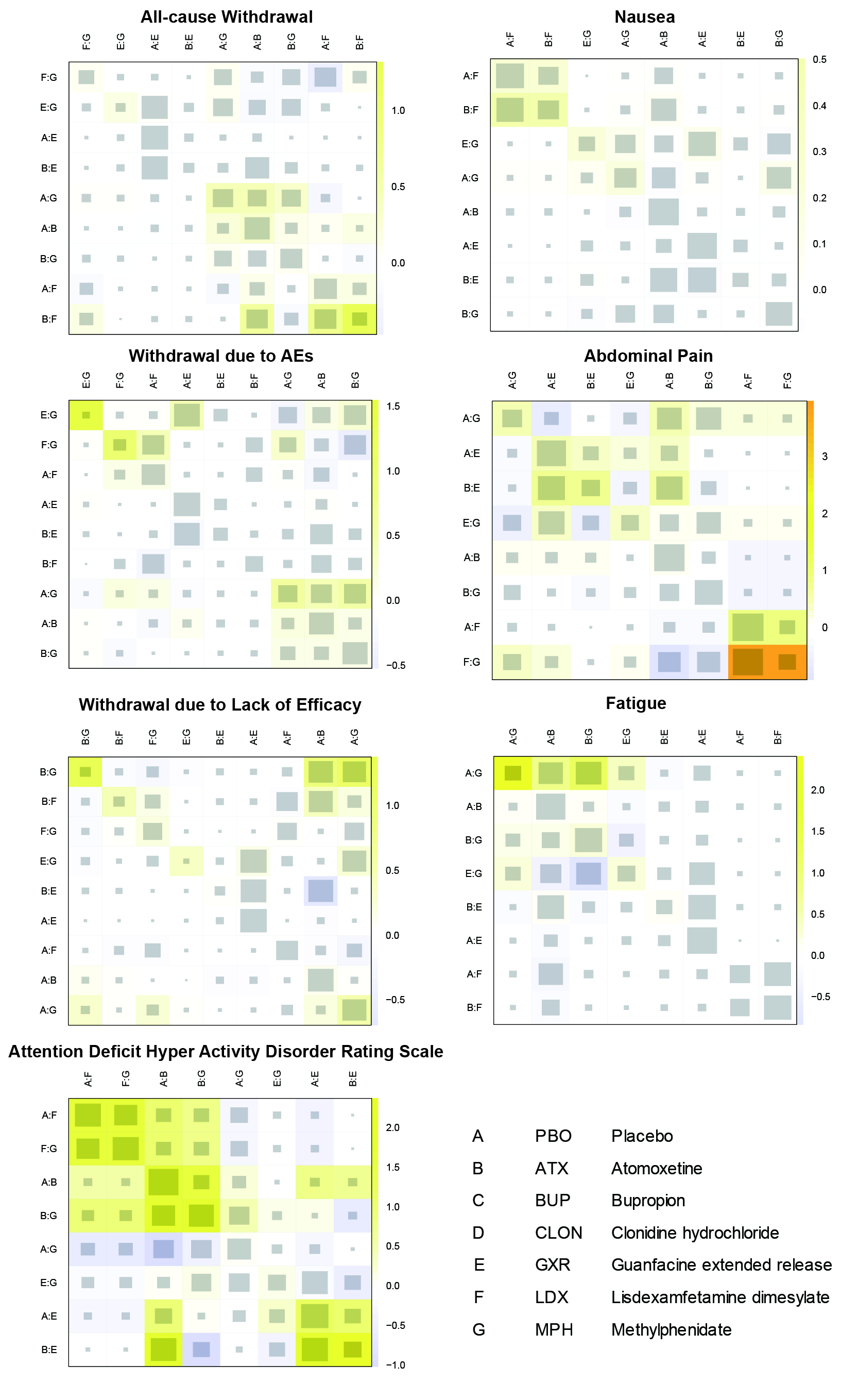

Supplement: Figure S2 — Heat plot. Consistency can be checked by contrasting effect estimates from direct comparisons with indirect evidence of all clinical outcomes. In combination, we show heat colors corresponding to the change in agreement between direct and indirect estimate. The blue color means the two data possess good consistency while red color indicates inconsistency. [file Image_2.TIF]
